# Support of personalized medicine through risk-stratified treatment recommendations - an environmental scan of clinical practice guidelines

**DOI:** 10.1186/1741-7015-11-7

**Published:** 2013-01-09

**Authors:** Tsung Yu, Daniela Vollenweider, Ravi Varadhan, Tianjing Li, Cynthia Boyd, Milo A Puhan

**Affiliations:** 1 Department of Epidemiology, Johns Hopkins Bloomberg School of Public Health, Baltimore, MD 21205, USA; 2 Department of Medicine, Division of General Internal Medicine, Johns Hopkins School of Medicine, Baltimore, MD 21205, USA; 3 Department of Medicine, Division of Geriatric Medicine and Gerontology, Johns Hopkins School of Medicine, Baltimore, MD 21205, USA

**Keywords:** Cancer, cardiovascular disease, chronic disease, COPD, diabetes, guidelines, randomized trials, risk assessment, stroke, treatment

## Abstract

**Background:**

Risk-stratified treatment recommendations facilitate treatment decision-making that balances patient-specific risks and preferences. It is unclear if and how such recommendations are developed in clinical practice guidelines (CPGs). Our aim was to assess if and how CPGs develop risk-stratified treatment recommendations for the prevention or treatment of common chronic diseases.

**Methods:**

We searched the United States National Guideline Clearinghouse for US, Canadian and National Institute for Health and Clinical Excellence (United Kingdom) CPGs for heart disease, stroke, cancer, chronic obstructive pulmonary disease and diabetes that make risk-stratified treatment recommendations. We included only those CPGs that made risk-stratified treatment recommendations based on risk assessment tools. Two reviewers independently identified CPGs and extracted information on recommended risk assessment tools; type of evidence about treatment benefits and harms; methods for linking risk estimates to treatment evidence and for developing treatment thresholds; and consideration of patient preferences.

**Results:**

We identified 20 CPGs that made risk-stratified treatment recommendations out of 133 CPGs that made any type of treatment recommendations for the chronic diseases considered in this study. Of the included 20 CPGs, 16 (80%) used evidence about treatment benefits from randomized controlled trials, meta-analyses or other guidelines, and the source of evidence was unclear in the remaining four (20%) CPGs. Nine CPGs (45%) used evidence on harms from randomized controlled trials or observational studies, while 11 CPGs (55%) did not clearly refer to harms. Nine CPGs (45%) explained how risk prediction and evidence about treatments effects were linked (for example, applying estimates of relative risk reductions to absolute risks), but only one CPG (5%) assessed benefit and harm quantitatively and three CPGs (15%) explicitly reported consideration of patient preferences.

**Conclusions:**

Only a small proportion of CPGs for chronic diseases make risk-stratified treatment recommendations with a focus on heart disease and stroke prevention, diabetes and breast cancer. For most CPGs it is unclear how risk-stratified treatment recommendations were developed. As a consequence, it is uncertain if CPGs support patients and physicians in finding an acceptable benefit- harm balance that reflects both profile-specific outcome risks and preferences.

## Background

An important goal of evidence-based health care is to maximize benefits and minimize harms from medical treatments. To achieve an optimal balance, patients' individual profiles and preferences need to be considered [1]. For example, inhaled corticosteroids are used to prevent exacerbations in patients with chronic obstructive pulmonary disease (COPD) [2-4], but these drugs are associated with an increased risk for pneumonia and fractures [5,6]. In patients at high risk for exacerbations, the potential benefits (preventing exacerbations) are likely to be larger than harms, while patients at low risk for exacerbations may experience more harms from inhaled corticosteroids than benefits.

Risk-stratified treatment recommendations are potentially useful to support personalized medicine. Personalized medicine aims at optimizing the benefit-harm balance by considering patient profiles (combination of characteristics) and preferences [7]. For the prevention and treatment of chronic disease, most health care decisions are sensitive to patient profiles and preferences [8]. Risk-stratified treatment recommendations suggest different treatment regimens for patients who are at different risks for outcomes [9]. For example, in the Third Report of the National Cholesterol Education Program's Adult Treatment Panel treatment algorithm [10], the recommendation for primary prevention of coronary heart disease is based on the Framingham Risk Score. According to different risk categories predicted by the Framingham Risk Score, individuals with higher predicted absolute risk (10-year risk > 20%) are recommended for more intensive treatments (such as combined pharmacological and non-pharmacological treatments) than those with lower predicted risk (10-year risk < 10%). There is evidence that using risk-stratified treatments is superior to treatments that are not informed by a risk assessment tool [11-13].

Risk-stratified treatment recommendations only serve their purpose of supporting personalized medicine if valid methods were used to develop them. Because it is not known what proportion of clinical practice guidelines (CPGs) make risk-stratified treatment recommendations and what methods were used to develop them, our aim was to assess the methods CPGs applied in developing risk-stratified treatment recommendations for the prevention or treatment of selected common chronic diseases.

## Methods

### Framework for developing risk-stratified treatment recommendations

**Figure 1:**
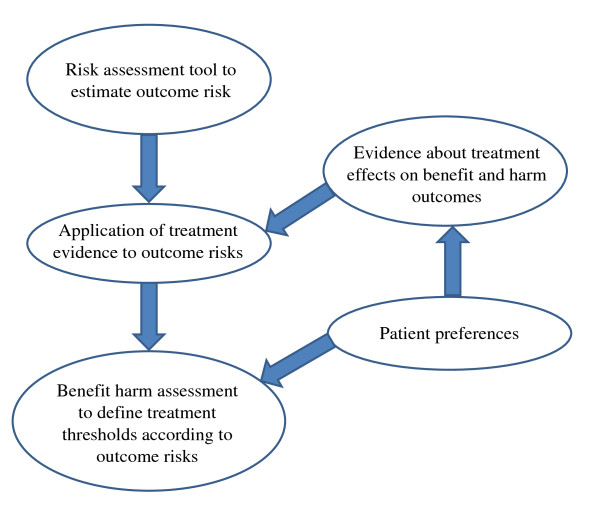
**Important elements for the development of risk-stratified treatment recommendations**.

We started by forming a framework for developing risk-stratified treatment recommendations. Figure 1 outlines the major steps for developing risk-stratified treatment recommendations, each of which requires high quality evidence from observational studies (development and validation of risk assessment tools), randomized trials (evidence about treatment effects) and studies to elicit patient preferences (using various study designs, for example, discrete choice experiments). It is well known for all guidelines that evidence about treatment effects on benefit and harm outcomes must be available. In addition, a risk assessment tool should be available that allows the assigning of patients to different risk categories. A method is required to estimate how treatment evidence applies to patients at different risks and how the benefits compare to the harms in patients at different risks. As a result of such a benefit-harm assessment, treatment thresholds can be defined for patients with different risk profiles that maximize the chance for benefits while minimizing harms. In addition, patient preferences for outcomes would ideally be explicitly considered for the development of risk-stratified treatment recommendations or their application in practice.

### Environmental scan of clinical practice guidelines

We performed an environmental scan of CPGs, which included a limited literature search (described below) but not a comprehensive, systematic review of all CPGs. We focused on CPGs for major chronic diseases and from the United States (US), Canada, or the United Kingdom (UK) National Institute for Health and Clinical Excellence (NICE). The completed PRISMA checklist is available as Additional file 1.

### Data sources and searches

We searched the US National Guideline Clearinghouse (NGC) database on February 5 2011 for CPGs with treatment recommendations for five major chronic diseases. The top five chronic diseases in the US are heart disease, cancer, stroke, COPD and diabetes, accounting for more than two-thirds of all deaths [14]. In the NGC database, guidelines were categorized by disease topics that were linked to a specific term derived from the US National Library of Medicine's Medical Subject Headings classification.

For heart disease and stroke, we performed our search within the *Cardiovascular Diseases *section of the database (n = 442) and considered CPGs specific for primary prevention of heart disease and stroke, that is, the prevention of an event in persons free of established cardiovascular diseases. For cancer, we chose to examine three types of cancer with the highest mortality rates in the US (lung cancer, prostate cancer and breast cancer) [15]. We searched for CPGs within the *Lung Neoplasms *(n = 53), *Prostatic Neoplasms *(n = 26) and *Breast Neoplasms *(n = 52) sections, respectively. For COPD, we considered CPGs specific for COPD within the *Respiratory Tract Diseases *section (n = 102). For diabetes mellitus, we considered CPGs for type II diabetes within the *Diabetes Mellitus, Type 2 *section (n = 44).

### Eligibility criteria for guidelines

We included CPGs that recommended using risk assessment tools to inform treatment decisions. Risk assessment tools are tools to calculate the probability of developing an event or a disease based on a prediction model (binary outcome), or tools that make projections about the course of disease measured by patient-reported or other continuous outcomes (for example, decline of functional status over time). We excluded CPGs if they were not from the US, Canada or NICE (UK); focused on childhood diseases; made recommendations on screening, genetic counseling or diagnostic work-up alone; or did not use any risk assessment tools to inform risk-stratified treatment decisions. This latter excluded category involved guidelines that recommended treatments according to diagnostic criteria, as for example based on pathological staging, rather than according to prognostic information (for example, the risk stratification scheme proposed by D'Amico *et al*. in prostate cancer guidelines [16]).

### Guideline selection

Two reviewers (TY and DV) independently reviewed the *Guideline Summary *section of each CPG on the NGC website to assess its potential eligibility. We excluded the CPGs labeled ineligible by both reviewers. For the other CPGs, we retrieved and examined the full text and resolved any discrepancies in eligibility through discussion or arbitration by a third reviewer (MP).

### Data extraction and synthesis

We developed a standardized form to extract data from the included CPGs and the background documents detailing the methods used in developing CPGs when available. We extracted general items such as guideline title, bibliographic source, date released and guideline developer. We then extracted information related to five key components for developing risk-stratified treatment recommendations (Figure 1). We extracted the following information on risk assessment tools: the name of the prediction model, the outcome and the timeframe (for example, 10 years) used in the model, and whether validation of the model (for example, assessment of discrimination and/or calibration) was discussed in the CPGs. We extracted information on the type of evidence used to determine the effects of treatments on benefit and harm outcomes (observational studies, single or several randomized controlled trials (RCTs), or meta-analyses). We recorded the methods to link risk prediction and evidence on treatment effects (for example, applying relative risk reductions to different absolute risks calculated from the risk assessment tool). We recorded the way the treatment benefits and harms were assessed and how treatment thresholds (based on risk assessment tools) were determined. We also extracted information on assumptions made for linking risk prediction and treatment evidence (for example, assumption of constant relative risk reductions across the risk spectrum) and on assumptions made for the assessment of benefits and harms (for example, assumption that benefit and harm outcomes can be put on a single scale and the overall net benefit expressed as a single number indicating benefit or harm). Finally, we noted whether patient preferences (for example, relative importance of different benefit and harm outcomes) were considered for developing risk-stratified treatment recommendations. Because some CPGs were very brief, without detailing the development process but referring to other documents, we considered those documents for data extraction to avoid underestimating the rigor of the development process of a CPG. Two reviewers (TY and DV) independently extracted all relevant information from each CPG and the discrepancies were resolved by discussion or third-party (MP) arbitration. We constructed a table for comparison of recommendations from each of the included CPGs.

## Results

**Figure 2:**
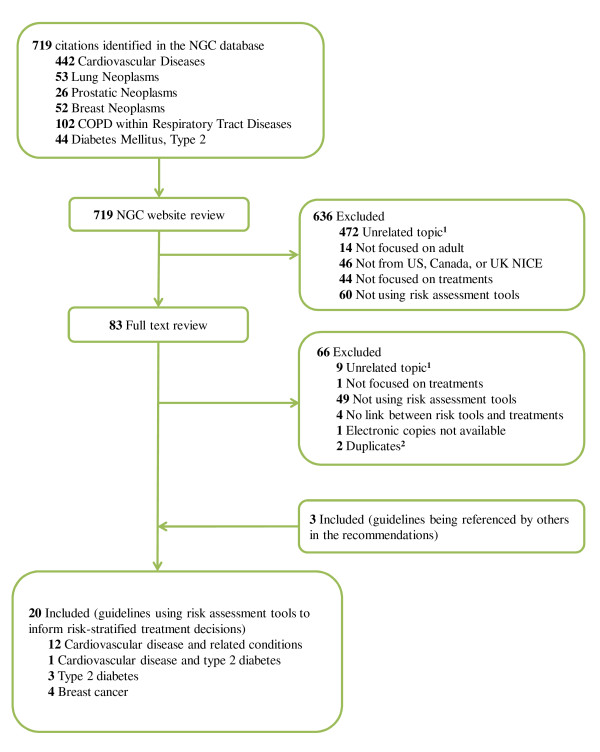
**Guideline search and review process**. ^1^For cardiovascular diseases, we excluded guidelines not focused on primary prevention. ^2^Two citations in *Diabetes Mellitus, Type 2 *were the same guidelines as in *Cardiovascular Diseases*. NGC: US National Guideline Clearinghouse; NICE: UK National Institute for Health and Clinical Excellence.

Most CPGs that we excluded (Figure 2) were on topics not related to our study question or because they were not from the US, Canada or NICE (UK). We excluded 60 CPGs based on NGC website review and 49 CPGs based on the full text because they did not recommend using a risk assessment tool. We excluded four additional CPGs because they recommended using a risk assessment tool but did not make any link to treatments (Figure 2). Thus out of 133 CPGs that made treatment recommendations for the chronic diseases of interest (60 + 49 + 4 + 20 = 133 CPGs), 20 made risk-stratified treatment recommendations (15%) for heart disease, stroke, type II diabetes or breast cancer (Figure 2) [10,17-36]. The characteristics of the 20 included CPGs are summarized in Table 1.

**Table 1:** Characteristics of the included guidelines.

Guideline identifier, year released	Guideline developer and country	Disease or condition	Guideline title
**Cardiovascular disease and related condition**			
**NCEP**, 2002 (updated 2004) [10,14]	National Heart, Lung, and Blood Institute, US	Hypercholesterolemia and coronary heart disease	National Cholesterol Education Program Expert Panel on Detection, Evaluation, and Treatment of High Blood Cholesterol in Adults (Adult Treatment Panel III)
**NICE1**, 2006 [15]	National Institute for Health and Clinical Excellence, UK	Cardiovascular disease	Statins for the Prevention of Cardiovascular Events
**AHA1**, 2006 [16]	American Heart Association and American Stroke Association, US	Ischemic stroke	Primary Prevention of Ischemic Stroke: A Guideline From the American Heart Association/American Stroke Association Stroke Council
**AHA2**, 2007 [17]	American Heart Association, US	Cardiovascular disease	Evidence-Based Guidelines for Cardiovascular Disease Prevention in Women: 2007 Update
**MSC1**, 2008 [18]	Medical Services Commission, British Columbia, Canada	Cardiovascular disease	Cardiovascular Disease - Primary Prevention
**NICE2**, 2008 [19]	National Collaborating Centre for Primary Care, UK	Cardiovascular disease	Lipid Modification. Cardiovascular Risk Assessment and the Modification of Blood Lipids for the Primary and Secondary Prevention of Cardiovascular Disease
**ACCP**, 2008 [20]	American College of Chest Physicians, US	Coronary artery disease	The Primary and Secondary Prevention of Coronary Artery Disease: American College of Chest Physicians Evidence-Based Clinical Practice Guidelines (8th Edition)
**MSC2**, 2008 [21]	Medical Services Commission, British Columbia, Canada	Hypertension	Hypertension - Detection, Diagnosis and Management
**USPSTF**, 2009 [22]	U.S. Preventive Services Task Force, US	Cardiovascular disease	Aspirin for the Prevention of Cardiovascular Disease: U.S. Preventive Services Task Force Recommendation Statement
**UMHS1**, 2009 [23]	University of Michigan Health System, US	Coronary heart disease and stroke	Screening and Management of Lipids
**ICSI**, 2009 [24]	Institute for Clinical Systems Improvement, US	dyslipidemia and coronary heart disease	Lipid Management in Adults
**MQIC**, 2009 [25]	Michigan Quality Improvement Consortium, US	Hypercholesterolemia	Screening and Management of Hypercholesterolemia
**Cardiovascular disease and type 2 diabetes**			
**ES**, 2008 [26]	The Endocrine Society, US	Cardiovascular disease and Type 2 diabetes	Primary Prevention of Cardiovascular Disease and Type 2 Diabetes in Patients at Metabolic Risk: An Endocrine Society Clinical Practice Guideline
**Type 2 diabetes**			
**NICE3**, 2008 [27]	National Clinical Guideline Centre for Acute and Chronic Conditions, UK	Type 2 diabetes	Type 2 Diabetes: National Clinical Guideline for Management in Primary and Secondary Care (update)
**MSC3**, 2010 [28]	Medical Services Commission, British Columbia, Canada	Type 2 diabetes	Diabetes Care
**ADA**, 2011 [29]	American Diabetes Association, US	Type 2 diabetes	Standards of Medical Care in Diabetes - 2011
**Breast cancer**			
**UMHS2**, 2007 [30]	University of Michigan Health System, US	Breast cancer	Common Breast Problems
**NSGC**, 2007 [31]	National Society of Genetic Counselors, US	Breast cancer and ovarian cancer	Risk Assessment and Genetic Counseling for Hereditary Breast and Ovarian Cancer: Recommendations of the National Society of Genetic Counselors
**ASCO**, 2009 [32]	American Society of Clinical Oncology, US	Breast cancer	American Society of Clinical Oncology Clinical Practice Guideline Update on the Use of Pharmacologic Interventions Including Tamoxifen, Raloxifene, and Aromatase Inhibition for Breast Cancer Risk Reduction
**NICE4**, 2009 [33]	National Collaborating Centre for Cancer, UK	Breast cancer	Early and Locally Advanced Breast Cancer. Diagnosis and Treatment

### Risk assessment tools used to estimate baseline risk for outcome of interest

A large proportion of CPGs (16 out of 20, 80%) were on type II diabetes or on primary prevention of heart disease and stroke [10,18-32], and the remaining four CPGs were on breast cancer management [33-36]. All 16 CPGs on diabetes or cardiovascular disease recommended one or several risk assessment tools to assess 10-year cardiovascular disease risk. The Framingham Risk Score was explicitly suggested in 12 CPGs [10,19-29]; the UK Prospective Diabetes Study Risk Engine was suggested in four CPGs [21,24,30,31] for patients with diabetes, and the Prospective Cardiovascular Münster and the Systematic Coronary Risk Evaluation risk tools were used in one CPG [29]. Of the 16 CPGs on diabetes or cardiovascular disease, two (13%) did not clearly specify the prediction model used for calculating 10-year cardiovascular disease risk [18,32]. Among the four CPGs on breast cancer [33-36], two recommended using the National Cancer Institute breast cancer risk assessment tool to calculate 5-year risk of invasive breast cancer [33,35]; one recommended using the Nottingham Prognostic Index to calculate 10-year survival [36]; one mentioned different risk assessment tools but did not clearly define risk categories [34]. Information on the validation of the risk models was reported in seven (35%) of the 20 included CPGs (Table 2) [10,19,20,22,29,30,35].

**Table 2:** Risk-stratified treatment recommendations of the included guidelines.

Guideline title	NCEP	NICE1	AHA1	AHA2
**Risk assessment tools**				
**Risk prediction model**	Framingham Risk Score	10-year risk of developing CVD, not referring to a specific risk model	Framingham Risk Score	Framingham Risk Score
**Outcome of interest and its timeframe**	CHD (10 years)	CVD (CHD and stroke, 10 years)	CHD (10 years)	CHD (10 years)
**Information on validation of the model provided in the guideline**	Yes	Unclear	Yes	Yes
**Evidence of treatment effects**				
**Treatment considered**	LDL-lowering therapy, therapeutic lifestyle change and LDL goals	Statin	Diet, weight management, physical activity, drug therapy and LDL-C goals	Lifestyle management, pharmacotherapy and LDL-C target levels
**Target population**	Adults	Adults at risk of CVD	Adult patients at increased risk of stroke	Adult women 20 years and older
**Type of studies considered in the evidence of treatment benefits**	Single or several RCTs Meta-analyses	Single or several RCTs Meta-analyses	Single or several RCTs Meta-analyses	Single or several RCTs Meta-analyses
**Type of studies considered in the evidence of treatment harms**	Observational studies Single or several RCTs	Single or several RCTs Meta-analyses	Treatment harms not reported	Treatment harms not reported
**Heterogeneity of treatment effects assessed in the guideline**	Yes	Yes	No	No
**Application of treatment evidence to baseline risks**				
**Methods to apply treatment evidence to baseline risks**	Used evidence of relative risk reduction from RCT/meta-analysis and applied it to different absolute risks	Unclear, presumably used evidence of relative risk reduction from RCT/meta-analysis and applied it to different absolute risks	Not reported	Not reported
**Assumptions specified when applying treatment evidence**	'For every 30-mg/dL change in LDL-C, the relative risk for CHD is changed in proportion by about 30%, and the relative risk is set at 1.0 for LDL-C = 40 mg/dL.' 'For every 1% reduction in LDL-C levels, relative risk for major CHD events is reduced by approximately 1%.'	'Statins do not differ in their relative effectiveness in a number of subgroups: in women compared with men at a similar level of cardiovascular risk; in people with diabetes compared with people without diabetes; or in people aged over 65 years compared with people aged under 65 years.'	Not reported	Not reported
**Development of treatment thresholds**				
**Risk stratification in which different treatments were recommended**	•10-year CHD risk > 20% •10-year CHD risk 10 to 20% •10-year CHD risk < 10%	•10-year CVD risk ≥20%	•0 to 1 CHD risk factor •≥2 CHD risk factors and 10-year CHD risk < 20% •≥2 CHD risk factors and 10-year CHD risk 10% to 20% •CHD or CHD risk equivalent (10-year risk > 20%)	•10-year CHD absolute risk > 20% •10-year CHD absolute risk 10% to 20% •10-year CHD absolute risk < 10%
**Methods to develop treatment thresholds**	Unclear	Expert consensus	Referring to NCEP ATP-III guideline	Not reported
**Explicitly planned benefit and harm assessment as the basis for making recommendations**	No	No	No	No
**Patient preferences considered when developing recommendations**	No	No	No	No

**Guideline title**	**MSC1**	**NICE2**	**ACCP**	**MSC2**

**Risk assessment tools**				
**Risk prediction model**	Framingham Risk Score (for patients without diabetes) and UKPDS Risk Engine (for patients with diabetes)	Framingham Risk Score	Framingham Risk Score	Framingham Risk Score or UKPDS Risk Engine for patients with diabetes
**Outcome of interest and its timeframe**	CHD (10 years)	CVD (CHD and stroke, 10 years)	CHD (10 years)	CHD (10 years)
**Information on validation of the model provided in the guideline**	No	Yes	No	No
**Evidence of treatment effects**				
**Treatment considered**	Lifestyle management, pharmacologic treatment and desirable lipid results	Lifestyle advice and statin	Aspirin and vitamin K antagonists	Lifestyle management and antihypertensive drugs
**Target population**	Men aged > 40 years and women aged > 50 years	Adults aged 18 and older and who have established CVD or who are at high risk of developing CVD	Patients at risk for coronary artery disease	Non-pregnant adults (age 19 years and older) with hypertension
**Type of studies considered in the evidence of treatment benefits**	Other guidelines	Meta-analyses	Meta-analyses	Meta-analyses
**Type of studies considered in the evidence of treatment harms**	Treatment harms not reported	Meta-analyses	Single or several RCTs Meta-analyses	Treatment harms not reported
**Heterogeneity of treatment effects assessed in the guideline**	No	Yes	Yes	No
**Application of treatment evidence to baseline risks**				
**Methods to apply treatment evidence to baseline risks**	Not reported	Not reported	Unclear, presumably used evidence of relative risk reduction from RCT/meta-analysis and applied it to different absolute risks	Used evidence of relative risk reduction from RCT/meta-analysis and applied it to different absolute risks
**Assumptions specified when applying treatment evidence**	Not reported	Not reported	Not reported	'This assumes 20% risk reduction of CHD based on average outcomes for appropriately used blood pressure lowering medications and statin medications.'
**Development of treatment thresholds**				
**Risk stratification in which different treatments were recommended**	•Framingham CHD risk ≥20% without CHD •Framingham CHD risk 10% to 19% •Framingham CHD risk < 10%	•CVD risk < 20% •CVD risk ≥20%	Moderate risk for a coronary event (10-year risk of a cardiac event > 10%)	Diagnosis of hypertension confirmed and CHD risk ≥20% over 10 years
**Method to develop treatment thresholds**	Referring to 2005 British Columbia guideline Diabetes Care	Referring to the NICE technology appraisal *Statins for the Prevention of Cardiovascular Events*	Unclear, presumably putting benefits and harms on the same scale and find a balance between them	Unclear
**Explicitly planned benefit and harm assessment as the basis for making recommendations**	No	Unclear	No	Unclear
**Patient preferences considered when developing recommendations**	No	No	No	No

**Guideline title**	**USPSTF**	**UMHS1**	**ISCI**	**MQIC**

**Risk assessment tools**				
**Risk prediction model**	Framingham Risk Score	Framingham Risk Score	Framingham Risk Score	Framingham Risk Score
**Outcome of interest and its timeframe**	CHD (10 years) in men and stroke (10 years) in women	Hard CHD (myocardial infarction and coronary death, 10 years)	CHD (10 years)	CHD (10 years)
**Information on validation of the model provided in the guideline**	No	No	No	No
**Evidence of treatment effects**				
**Treatment considered**	Aspirin	Lifestyle changes, drug therapy and LDL-C goals	Drug therapy and LDL goals	Drug therapy and goal for LDL-C
**Target population**	Men aged 45 to 79 years and women aged 55 to 79 years	Adults 20 to 75 years of age without familial or severe dyslipidemias	Adults 20 years and older and who are dyslipidemic	Adults ≥18 years
**Type of studies considered in the evidence of treatment benefits**	Meta-analyses	Treatment benefits reported but study type unclear	Single or several RCTs Meta-analyses	Treatment benefits not reported
**Type of studies considered in the evidence of treatment harms**	Observational studies	Treatment harms reported but study type unclear	Single or several RCTs Meta-analyses	Treatment harms not reported
**Heterogeneity of treatment effects assessed in the guideline**	Yes	Yes	No	No
**Application of treatment evidence to baseline risks**				
**Methods to apply treatment evidence to baseline risks**	Used evidence of relative risk reduction from RCT/meta-analysis and applied it to different absolute risks	Used evidence of relative risk reduction from RCT/meta-analysis and applied it to different absolute risks	Used evidence of relative risk reduction from RCT/meta-analysis and applied it to different absolute risks	Not reported
**Assumptions specified when applying treatment evidence**	There is 'a 32% risk reduction of MIs with regular aspirin use' (in men) and 'a 17% risk reduction of strokes with regular aspirin use' (in women). 'The risk for gastrointestinal bleeding increases with age.'	Not reported	Not reported	Not reported
**Development of treatment thresholds**				
**Risk stratification in which different treatments were recommended**	•Men aged 45 to 59 years and 10-year CHD risk ≥4%; men aged 60 to 69 years and 10-year CHD risk ≥9%; men aged 70 to 79 years and 10-year CHD risk ≥12% •Women aged 55 to 59 years and 10-year stroke risk ≥3%; women aged 60 to 69 years and 10-year stroke risk ≥8%; women aged 70 to 79 years and 10-year stroke risk ≥11%	•0 to 1 risk factors •2+ risk factors and 10-year CHD risk < 10% •2+ risk factors and 10-year CHD risk 10% to 20%	•0 to 1 risk factor and 10-year CHD risk < 10% •2+ risk factors and 10-year CHD risk < 10% •2+ risk factors and 10-year CHD risk 10% to 20% •CHD or CHD equivalent and/or 10-year risk > 20%	•CHD or CHD risk equivalents 10-year risk > 20% •2+ risk factors 10-year CHD risk ≤20% •0 to 1 risk factor
**Method to develop treatment thresholds**	Putting benefits and harms on the same scale (events saved/in excess per 1,000 people) and find a balance between them	Expert consensus and referring to NCEP ATP-III guideline	Referring to NCEP ATP-III guideline	Referring to ICSI Lipid Management in Adults guideline
**Explicitly planned benefit and harm assessment as the basis for making recommendations**	Yes	No	No	No
**Patient preferences considered for the development of recommendations**	Yes	No	No	No

**Guideline title**	**ES**	**NICE3**	**MSC3**	**ADA**

**Risk assessment tools**				
**Risk prediction model**	Framingham Risk Score, PROCAM and SCORE	UKPDS Risk Engine	UKPDS Risk Engine	Not specified, presumably Framingham Risk Score
**Outcome of interest and its timeframe**	10-year CHD risk (Framingham and PROCAM) and 10-year total cardiovascular mortality (SCORE)	CHD (10 years) in patients with diabetes	CHD (10 years) in patients with diabetes	CVD (CHD and stroke, 10 years)
**Information on validation of the model provided in the guideline**	Yes	Yes	No	No
**Evidence of treatment effects**				
**Treatment considered**	Aspirin, LDL-C goals and non-HDL-C goals	Simvastatin and statin	Statin and lipid targets	Aspirin
**Target population**	Patients at high metabolic risk for CVD	People with type 2 diabetes	Non-pregnant adults with type 2 diabetes	Patients with type 1 or type 2 diabetes mellitus
**Type of studies considered in the evidence of treatment benefits**	Single or several RCTs Meta-analyses	Single or several RCTs Meta-analyses	Single or several RCTs	Meta-analyses
**Type of studies considered in the evidence of treatment harms**	Treatment harms reported but study type unclear	Single or several RCTs	Treatment harms not reported	Treatment harms reported but study type unclear
**Heterogeneity of treatment effects assessed in the guideline**	No	No	No	Yes
**Application of treatment evidence to baseline risks**				
**Methods to apply treatment evidence to baseline risks**	Not reported	Not reported	Not reported	Unclear, presumably used evidence of relative risk reduction from RCT/meta-analysis and applied it to different absolute risks
**Assumptions specified when applying treatment evidence**	Not reported	Not reported	Not reported	Not reported
**Development of treatment thresholds**				
**Risk stratification in which different treatments were recommended**	•Individuals over age 40 and 10-year risk for CHD > 10% •10-year risk for CHD > 20% •10-year risk for CHD 10% to 20% •At least two major risk factors and 10-year risk for CHD < 10%	The cardiovascular risk exceeds 20% over 10 years	•Moderate risk (< 20% 10-year CHD risk) •High risk (≥20% 10-year CHD risk)	•Adults with type 1 or type 2 diabetes at increased cardiovascular risk (10-year CVD risk > 10%) •Adults with diabetes and 10-year CVD risk < 5% •Adults with 10-year CVD risk 5% to 10%
**Methods to develop treatment thresholds**	Unclear, presumably putting benefits and harms on the same scale and find a balance between them to recommend using aspirin; referring to NCEP ATP-III guideline on LDL-C and non-HDL-C goals	Not reported	Not reported	Unclear, presumably putting benefits and harms on the same scale and find a balance between them
**Explicitly planned benefit and harm assessment as the basis for making recommendations**	No	No	No	No
**Patient preferences considered when developing recommendations**	No	Yes	No	No

**Guideline title**	**UMHS2**	**NSGC**	**ASCO**	**NICE4**

**Risk assessment tools**				
**Risk prediction model**	NCI Breast Cancer Risk Assessment Tool	The guideline mentioned different models	NCI Breast Cancer Risk Assessment Tool	Nottingham Prognostic Index
**Outcome of interest and its timeframe**	Invasive breast cancer (5 years)	Absolute risk of developing breast cancer or the likelihood of carrying a *BRCA1 *or *BRCA2 *mutation (unclear timeframe)	Invasive breast cancer during the next 5-year period and up to age 90 (lifetime risk)	Survival (10 years)
**Information on validation of the model provided in the guideline**	No	No	Yes	No
**Evidence of treatment effects**				
**Treatment considered**	Tamoxifen and raloxifene	Tamoxifen; oral contraceptives; prophylactic mastectomy, prophylactic bilateral salpingo-oophorectomy	Tamoxifen and raloxifene	Aromatase inhibitors
**Target population**	Adults age 18 and older (non-pregnant)	Individuals at risk for hereditary breast and ovarian cancer	Women at increased risk of breast cancer	Women with breast cancer
**Type of studies considered in the evidence of treatment benefits**	Treatment benefits reported but study type unclear	Treatment benefits reported but study type unclear	Single or several RCTs Meta-analyses	Single or several RCTs
**Type of studies considered in the evidence of treatment harms**	Treatment harms reported but study type unclear	Treatment harms not reported	Single or several RCTs Meta-analyses	Single or several RCTs
**Heterogeneity of treatment effects assessed in the guideline**	No	No	Yes	No
**Application of treatment evidence to baseline risks**				
**Methods to apply treatment evidence to baseline risks**	Used evidence of relative risk reduction from the same risk profile population for which the recommendation was made	Not reported	Unclear	Unclear
**Assumptions when applying treatment evidence**	Not reported	Not reported	Not reported	Not reported
**Development of treatment thresholds**				
**Risk stratification in which different treatments were recommended**	Women at high risk (5-year risk of invasive cancer ≥1.7%)	The guideline made risk-stratified recommendations, but it is unclear how they defined high risk, moderate risk and low risk	Premenopausal and postmenopausal women with a 5-year projected breast cancer risk ≥1.66% or with lobular carcinoma *in situ*	•Postmenopausal women with estrogen-receptor-positive early invasive breast cancer not at low risk (those in the Excellent Prognosis Group or Good Prognosis Group in the Nottingham Prognostic Index) •Postmenopausal women with estrogen-receptor-positive early invasive breast cancer not at low risk and who have been treated with tamoxifen for 2 to 3 years
**Method to develop treatment thresholds**	Expert consensus	Not reported	Expert consensus	Unclear
**Explicitly planned benefit and harm assessment as the basis for making recommendations**	No	No	Yes	No
**Patient preferences considered when developing recommendations**	No	No	No	Yes

CHD: coronary heart disease; CVD: cardiovascular disease; LDL: low-density lipoprotein; LDL-C: low-density lipoprotein cholesterol; MI: myocardial infarction; NCEP ATP-III: National Cholesterol Education Program Adult Treatment Panel III; NCI: National Cancer Institute; NICE: National Institute for Health and Clinical Excellence; HDL-C: high-density lipoprotein cholesterol; PROCAM: Prospective Cardiovascular Münster; RCT: randomized clinical trial; SCORE: Systematic Coronary Risk Evaluation; UKPDS: United Kingdom Prospective Diabetes Study.

### Treatments recommended and evidence of treatment benefits and harms

Of the 16 CPGs for type II diabetes and primary prevention of heart disease and stroke, nine (56%) suggested specific target lipid levels for each risk category when making recommendations about lifestyle management or pharmacotherapy (for example, aspirin, statins and antihypertensive drugs) [10,19-21,26-29,31]. The four CPGs on breast cancer [33-36] provided recommendations on surgery or pharmacotherapy (for example, tamoxifen, raloxifene and aromatase inhibitors) according to risk levels.

Sixteen (80%) of the 20 CPGs reported using evidence on treatment benefits from RCTs or meta-analyses or other guidelines [10,18-25,27,29-32,35,36]. One CPG (5%) did not report quantitative information on treatment benefits [28] and three (15%) did not clearly specify the type of studies considered [26,33,34]. Treatment harms were only reported in 13 of the 20 CPGs (65%) [10,18,22,23,25-27,29,30,32,33,35,36]. The source of evidence on harms was specified in nine out of these 13 CPGs (69%) and included observational studies, RCTs and meta-analyses [10,18,32,23,25,27,30,35,36]. Heterogeneity of treatment effects were assessed in eight (40%) of the included 20 CPGs (Table 2) [10,18,22,23,25,26,32,35].

### Linking treatment effects to baseline risks

In reviewing how CPGs made the link between risk prediction and treatment effects, we found fewer than half of the CPGs (eight out of 20, 40%) explicitly or implicitly stated that they applied evidence of relative risk reductions from RCTs and/or meta-analyses to different absolute risks [10,18,23-27,32]. For instance, the U.S. Preventive Services Task Force (USPSTF) guideline [25] applied a 32% risk reduction of myocardial infarction (in men) and a 17% risk reduction of strokes (in women) with regular aspirin use to absolute outcome risks and assumed that the effects were constant across risk levels and age categories. One (5%) of the 20 included CPGs [33], instead of applying treatment evidence to all risk levels, used the evidence from RCTs with the same risk profile (high breast cancer risk) population for which the recommendation was made. Eleven (55%) of the included CPGs did not report the way in which they linked risk prediction to treatment effects (Table 2) [19-22,28-31,34-36].

### Benefit-harm assessment to define treatment thresholds and consideration of patient preferences

Only a small proportion (two out of 20, 10% [25,35]) of CPGs explicitly stated that they planned to perform benefit and harm assessment as the basis for making risk-stratified treatment recommendations. To define treatment thresholds, only the USPSTF guideline quantitatively weighed treatment benefits and harms by putting the expected benefit and harm outcomes on the same scale (events per 1,000 persons treated over 10 years). The USPSTF guideline recommended using aspirin when the treatment benefits (number of myocardial infarctions or strokes prevented per 1,000 persons treated over 10 years) outweigh the treatment harms (number of gastrointestinal bleedings or hemorrhagic strokes per 1,000 persons treated over 10 years). For example, the expected number of myocardial infarctions prevented by aspirin was estimated to be 16 per 1,000 men of age 60 to 69 years if men had a 10-year risk for myocardial infarction of 5%, while the expected number of excess gastrointestinal bleedings was 24 and hemorrhagic strokes was one. Because the number of excess events exceeded the number of prevented myocardial infarctions, the USPSTF recommended against the use of aspirin in men at 5% risk for myocardial infarction and an age of 60 to 69 years. Based on observational studies, the USPSTF assumed different risks for gastrointestinal bleeding with aspirin according to age. Finally, the USPSTF presented their benefit-harm assessment and the resulting treatment thresholds as a matrix table with categories for age and risk for myocardial infarction defining each cell.

Three (15%) of the 20 CPGs qualitatively weighed the treatment benefits and harms [23,29,32]. Nine (45%) of the 20 CPGs made the recommendation on thresholds based on expert consensus or referred to other guidelines [18,19,21,22,26-28,33,33]. Seven (35%) of the 20 CPGs did not report how they determined the treatment thresholds when making recommendations [10,20,24,30,31,34,36]. With regard to involving patient preferences when developing treatment recommendations, only three (15%) of the 20 CPGs explicitly reported that they considered patient preferences in the process (Table 2) [25,30,36]. For example, the USPSTF focused on major benefit (myocardial infarction) and harm events (gastrointestinal bleeding and hemorrhagic stroke) and assumed equal preferences (that is, importance) for those outcomes.

## Discussion

We found a rather small proportion of CPGs for heart disease, cancer, stroke, COPD and diabetes that made risk-stratified treatment recommendations using risk assessment tools. Most of these CPGs recommend risk assessment tools that had been shown to accurately predict outcome risk in the target population of the CPGs and most of the treatment evidence is based on RCTs and meta-analyses. For the majority of the CPGs, however, it was not explicitly explained how treatment effects on benefit and harm outcomes were estimated for patients at different risks. Perhaps most importantly, it was unclear for all but one CPG how treatment thresholds were determined to generate risk-stratified treatment recommendations.

We formed a framework for the development of risk-stratified treatment recommendations (Figure 1) to systematically identify the strengths and weaknesses of current CPGs. Our findings suggest that risk assessment tools were carefully appraised and selected during the development of CPGs. For example, some CPG developers critically appraised validation studies of risk tools to judge their calibration (agreement between predicted and observed risk) and discrimination (probability that those with an event receive higher risk predictions that those without an event) [10,30]. Minimizing misclassification of outcome risks is important to avoid over- or under-treatment [37-39]. While some CPGs recommended specific risk assessment tools, one CPG suggested using the risk assessment tool that is most likely to be accurate in the specific population of interest [30]. However, the set of CPGs selected in this study may give an overoptimistic picture of risk assessment tools proposed by guidelines. For many diseases and geographical locations other than the US, Canada and the UK, calibrated and discriminative risk assessment tools may not exist. A strength of existing CPGs is that the majority of them relied on RCTs and meta-analyses of RCTs for intervention effectiveness. The CPG developers recognized limitations within this body of evidence, including insufficient evidence on treatment heterogeneity (that is, subgroup effects) and scarcity of data on harm outcomes.

We discovered a number of major limitations in how CPGs develop risk-stratified treatment recommendations. It should be noted that some limitations propagated from single, prominent CPG (for example, National Cholesterol Education Program) to other CPGs that adopted the approach or even the recommendations. For example, it was often unclear how the benefit and harm outcomes were estimated for different risk profiles. Some CPGs applied estimates on relative risk reduction to absolute risks. This approach relies on the assumption of constant (relative) effects across the risk spectrum. This assumption of constant relative treatment effects may be justifiable in many instances but it is usually difficult to verify. No alternative approaches for linking the absolute risk with treatment evidence were used. Additional sensitivity analyses may sometimes be appropriate to explore the assumption of relative treatment effects. For example, one could obtain risk-specific treatment estimates from large trials using individual patient data [12]. Or, one could employ simulation studies to estimate the probability of outcomes in the population of interest by combining observational data and treatment effects from randomized trials. It is currently unclear what the most appropriate approach is to link risk predictions with evidence from randomized trials. Nevertheless, we believe CPGs should be explicit about the method they use and acknowledge the associated advantages and limitations (for example, assumption of constant relative risk reduction).

In our view, the greatest limitation of current CPGs is that it is unclear how treatment thresholds were developed for most of them. Some CPGs stated that the thresholds were determined by experts. The USPSTF guideline on aspirin [25] was the only guideline that conducted a formal quantitative assessment by comparing the expected number of benefit and harm events for patients at different risk for myocardial infarction and major gastrointestinal bleeding. We believe that transparency will be enhanced by conducting quantitative benefit-harm assessments alongside more qualitative approaches, such as using expert consensus about treatment thresholds.

Treatment thresholds are important because medical decision-making is discrete (to treat the patient or not). It is challenging to determine thresholds because clear cuts on the (commonly) continuous benefit-harm scale may not exist. In addition, there may often be substantial uncertainty about harms and heterogeneity of treatment effects as a consequence of poor reporting or a lack of evidence from primary studies. However, this should, in our view, not prevent CPG developers from making risk-stratified recommendations because health care providers need evidence-based guidance nevertheless and because variability in delivering health care may be unacceptably high in the absence of guidance. Quanstrum and Hayward [40] recently suggested an approach that acknowledges uncertainty about treatment decision thresholds and proposed two thresholds instead of one: one above which physicians should recommend treatments (benefits outweighing harms irrespective of patient preferences and uncertainties about evidence base) and one below which physicians should recommend against treatments (harms outweighing benefits). The interval between the two thresholds represents an area where treatment could provide small benefits or harms depending on patient preferences but also where uncertainty about the evidence precludes CPG developers from making recommendations. Alternatively, CPG developers could frame strong recommendations for or against treatment for patients at outcomes risks above or below the two thresholds, respectively, and weak recommendations for patients at outcome risks between the two thresholds [41].

One may criticize the approach used by the USPSTF, assigning equal weight to benefit and harm outcomes to calculate events expected per 1,000 people treated over 10 years, because empirical evidence suggests that patients, on average, assign different importance to myocardial infarction, major gastrointestinal bleeding and major stroke, the major drivers of the benefit-harm balance of aspirin [42]. Nevertheless, such transparency about the relative importance of outcomes comes with several important advantages. Users of CPGs can understand and replicate how the treatment thresholds were derived and, if they do not agree with certain assumptions (for example, equal importance of myocardial infarction and major gastrointestinal bleeding), they can adjust the result to derive thresholds that would suit their settings (for example, myocardial infarction considered twice as important as major gastrointestinal bleeding). This would also allow the guideline to be interpreted for an individual patient, who may weigh the various outcomes differently than those preferences assumed in the CPG.

The framework for developing risk-stratified treatment recommendation we proposed may be useful for those developing CPGs and to stimulate further research. While much research has been done on how to select and appraise evidence on treatment benefits and harms [43,44] and how to judge the validity of prediction models [37-39], it is less clear how to link risk prediction and treatment evidence, how to select a method for benefit-harm assessment to develop treatment thresholds, and how to include patient preferences. It would be useful to have empirical evidence on how the results of different approaches for linking risk prediction and treatment evidence and for defining treatment thresholds differ and how sensitive they are to assumptions [45]. As for patient preferences, little research has been done to find ways to include stakeholders in the process of selecting important outcomes, or a benefit-harm assessment method that provides the information patients need in order to make decisions [46-48]. The newly founded Patient-Centered Outcomes Research Institute is likely to contribute substantially to the questions raised.

Our study has some weaknesses. We selected guidelines from five major disease categories and from one database and focused on CPGs from the US, Canada and NICE (UK). Thus our results may not be generalizable, but provide an optimistic assessment of CPGs because we included some of the most prominent guidelines in medicine. For the fields of cardiovascular medicine and diabetes, guideline developers have a long tradition of making risk-stratified treatment recommendations. We relied on published reports, which may not reflect the true underlying development process for CPGs. We considered all background documents that were openly accessible but we may have missed some information on the development of risk-stratified treatment recommendations.

## Conclusions

We found that the methods for linking risk prediction with treatment evidence are often not reported and that it was unclear for all but one CPG how treatment thresholds were developed. Therefore, current CPGs for major chronic diseases may not support patients and physicians in finding an acceptable benefit-harm balance that reflects profile-specific outcome risks and preferences.

## Abbreviations

COPD: chronic obstructive pulmonary disease; CPG: clinical practice guideline; NGC: National Guideline Clearinghouse; NICE: National Institute for Health and Clinical Excellence; RCT: randomized controlled trial; USPSTF: U.S. Preventive Services Task Force.

## Competing interests

The authors declare that they have no competing interests.

## Authors' contributions

All authors contributed to the conception of the study; TY, DV and MP collected and analyzed the data; all authors interpreted the data; TY and MP drafted the article; all authors revised it critically for important intellectual content; and all authors read and approved the final manuscript.

## Pre-publication history

The pre-publication history for this paper can be accessed here:

http://www.biomedcentral.com/1741-7015/11/7/prepub

## Supplementary Material

Additional file 1 **PRISMA checklist**. The additional file shows the completed PRISMA checklist and shows where the checklist items are reported. Click here for file
